# Supplementary figures and images for: A mixture of sparse coding models explaining properties of face neurons related to holistic and parts-based processing
Source: PLoS Comput Biol. 2017 Jul 25;13(7):e1005667. doi: 10.1371/journal.pcbi.1005667 (PMC5549761; doi:10.1371/journal.pcbi.1005667)

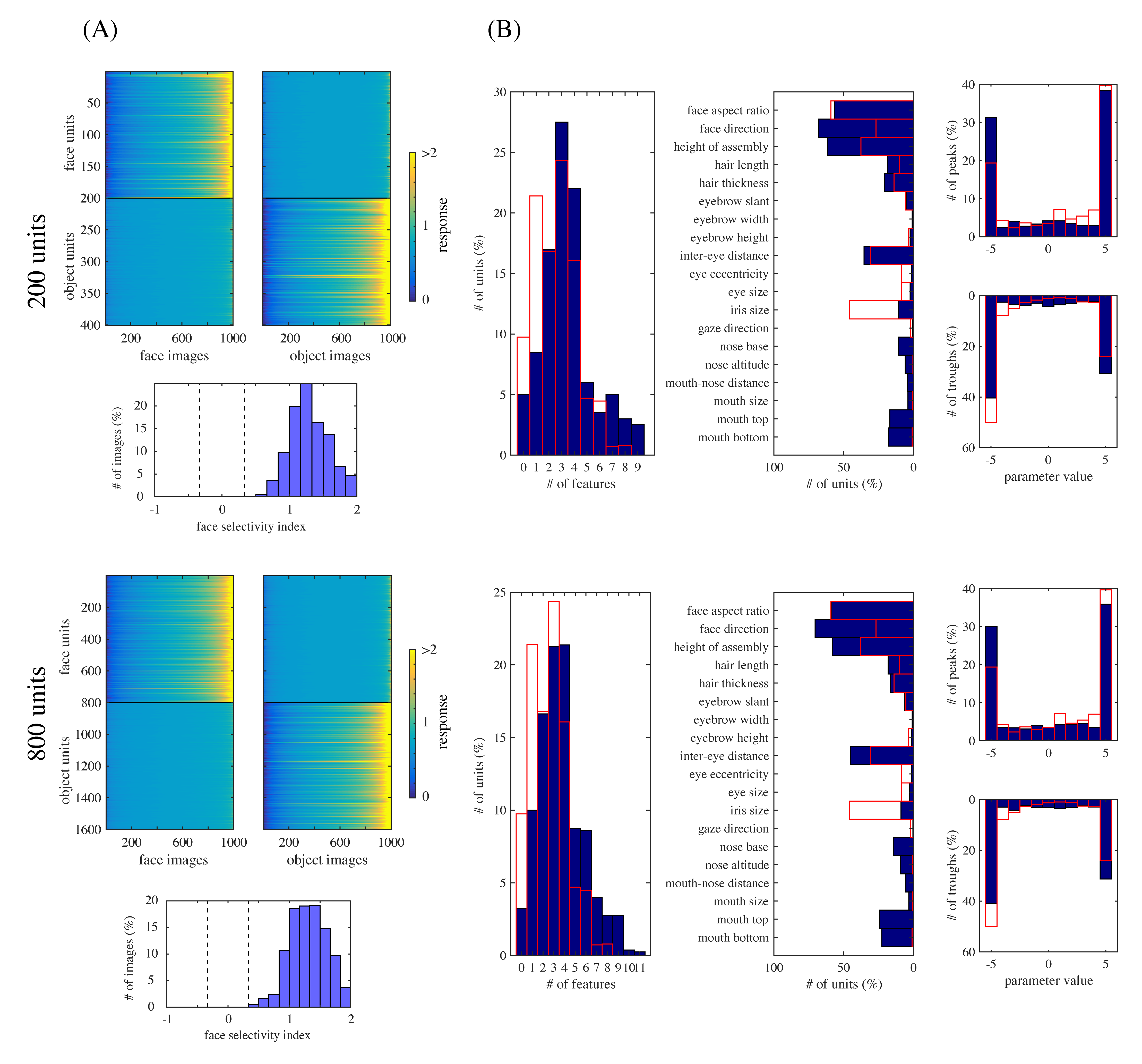

Supplement: S1 Fig — A mixture model was constructed in the same way as the original one, except that each submodel here had 200 units (upper half) or 800 units (lower half). (A) The responses of model face units and object units to natural face images (left) or natural object images (right), together with the distribution of face-selective indices for the face units (bottom); compare these with Fig 4A and 4D (blue). (B) The distributions of the numbers of significantly tuned features (of cartoon faces) per unit (left), of numbers of significantly tuned units for each feature parameter (middle), of peak and trough parameter values (right); compare these with Figs 6 and 7B. Overlaid red boxes are replots of corresponding experimental data [4]. (TIF) [file pcbi.1005667.s001.tif]

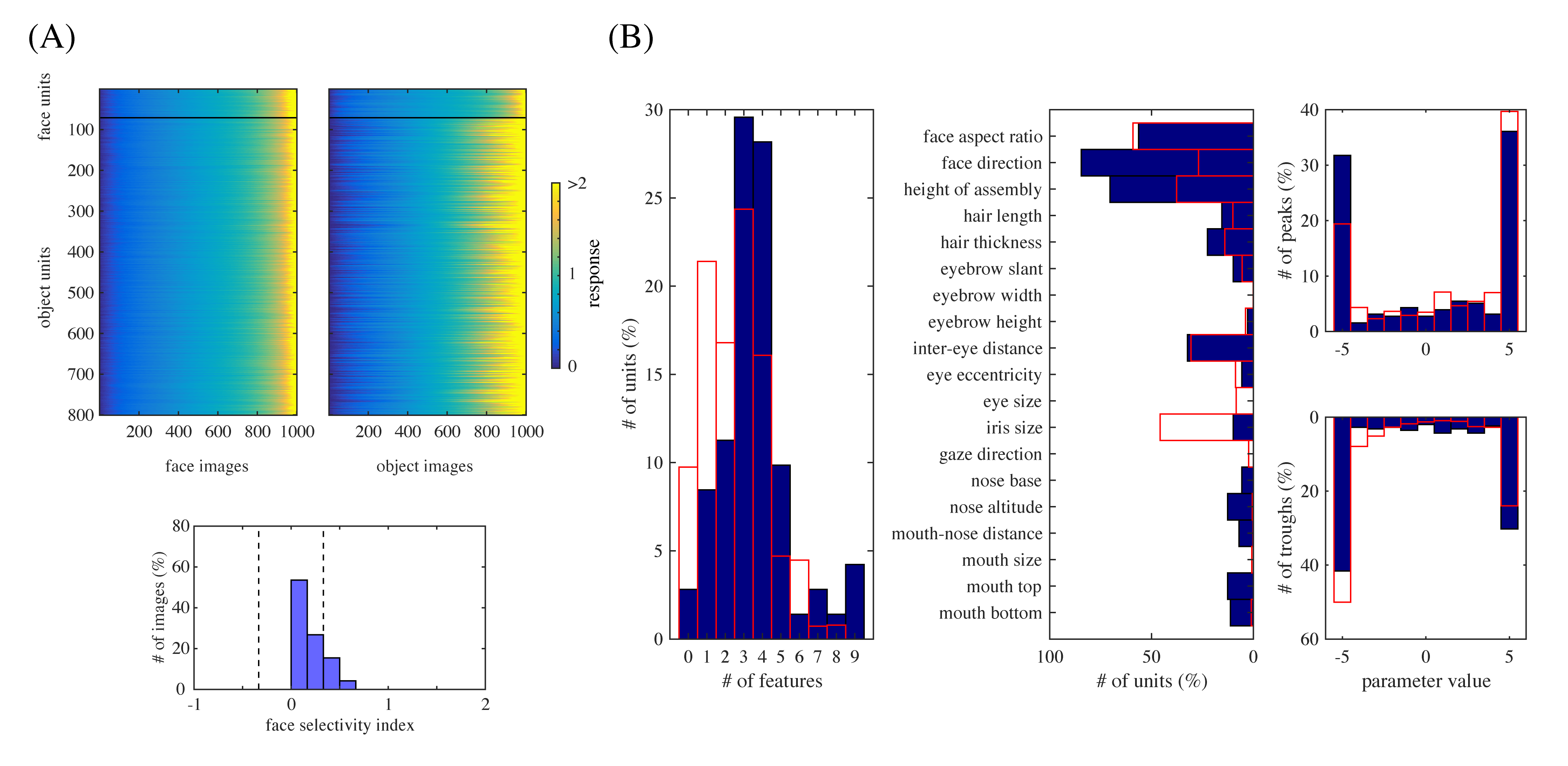

Supplement: S2 Fig — A single sparse coding model with 800 units was constructed on top of the same energy model and trained with an ensemble of face and non-face images. In the resulting model, only 71 units gave larger average responses to face images than non-face images. The response properties of these units are shown. (A) The responses of face and object units to face images (left) or object images (right), with the distribution of face-selective indices for the face units (bottom). No prominent selectivity like in Fig 4A can be observed; the result is more similar to Fig 4B. (B) The distributions of the numbers of significantly tuned features per unit (left), of numbers of significantly tuned units for each cartoon face feature parameter (middle), of peak and trough parameter values (right); compare these with Figs 6 and 7B as well as Fig 10 (cyan curves). Overlaid red boxes are replots of corresponding experimental data [4]. (TIF) [file pcbi.1005667.s002.tif]

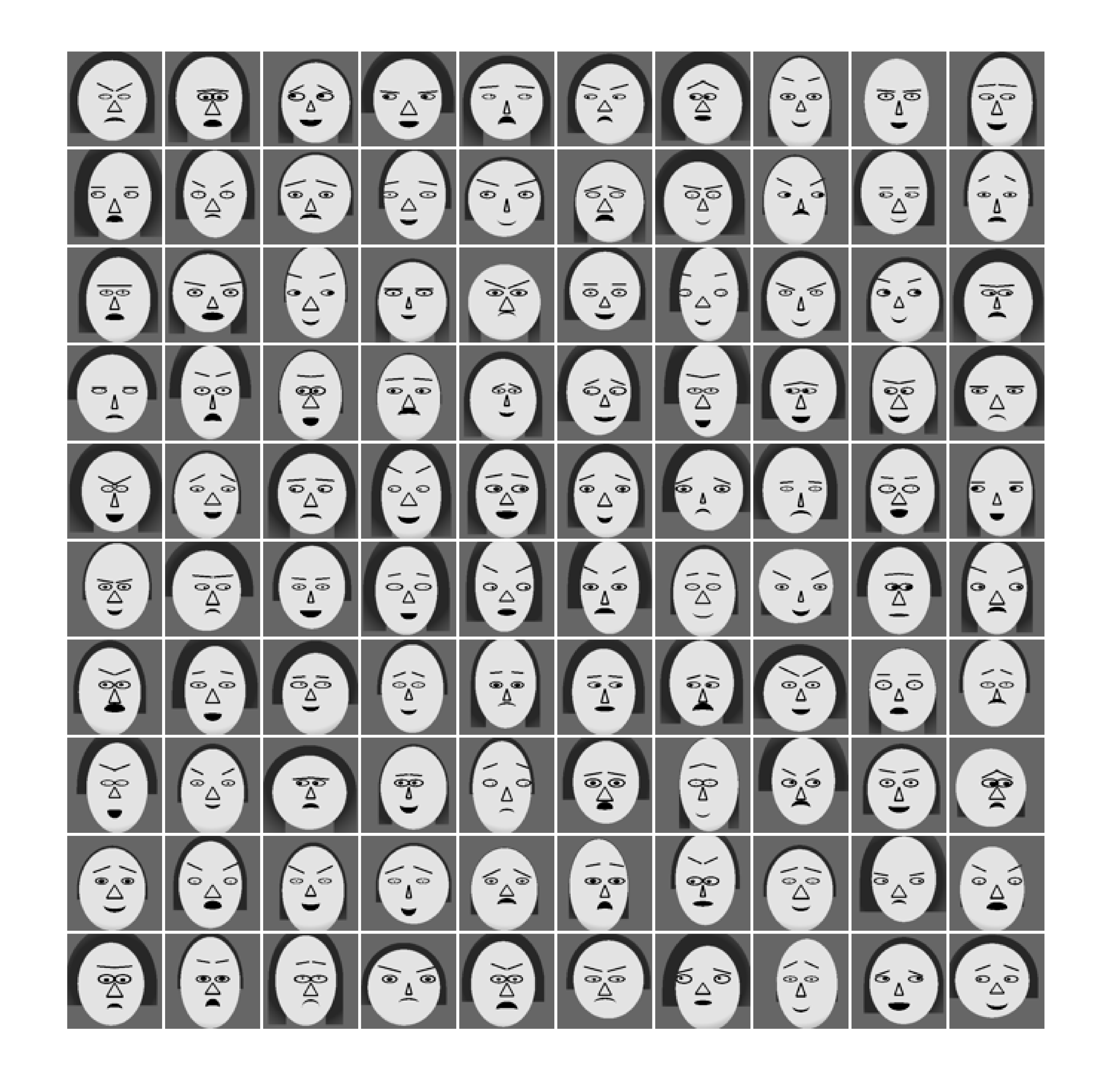

Supplement: S3 Fig — (TIF) [file pcbi.1005667.s003.tif]
